# In Memoriam: Michael B. Gregg (1930–2008)

**DOI:** 10.3201/eid1409.080952

**Published:** 2008-09

**Authors:** David M. Morens

**Affiliations:** National Institutes of Health, Bethesda, Maryland, USA

**Keywords:** obituary, Michael B. Gregg, commentary

**Figure:**
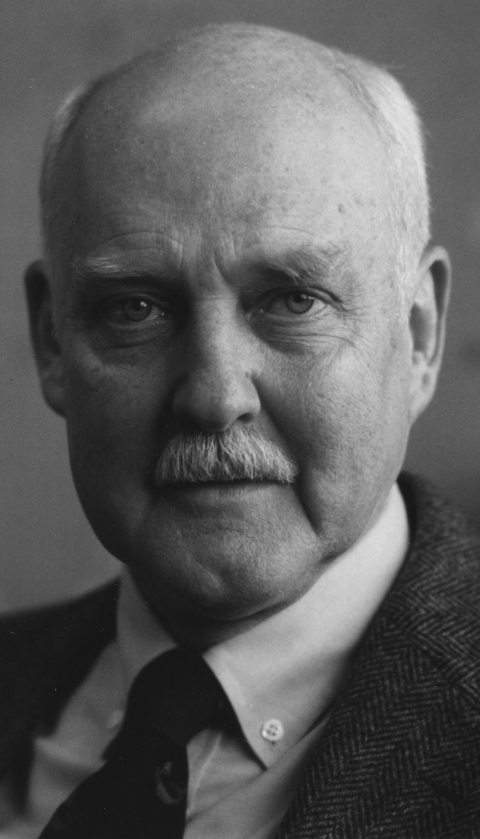
Dr Gregg

“He has in his make-up two essential elements—common sense and the will to work ... in collecting [facts] he is diligent, patient, careful, thorough and unbiased ... he avoids needless or obfuscating high mathematics and formulae ... he does not twist the facts... He remains broad-minded and open-minded ... He is humble in his ignorance but bold in his search for truth” (*1*). So wrote legendary American epidemiologist Leslie Lumsden (1875–1946). He was responding to the question, posed by the American Journal of Public Health in 1942, “What and who is an epidemiologist?” (*1*).

Michael B. Gregg, MD, who died on July 9 in Brattleboro, Vermont, was a 12-year-old boy in 1942 when Lumsden described the quintessential epidemiologist. But Gregg (or Mike, as just about everyone at the Centers for Disease Control and Prevention [CDC] called him) grew up to become not only an epidemiologist but also a teacher, a mentor, a friend, and for many the embodiment of Lumsden’s ideal (Figure). Among the many hundreds of students Gregg taught and influenced at the National Institutes of Health, and most notably at CDC, are countless leaders in epidemiology, public health, and the biomedical sciences. They practice all over the country, in health departments, in universities, in federal agencies, and indeed around the world. Roger Bernier, an epidemiologist who trained under Gregg at CDC, has compared Gregg’s professional approach to one of his hobbies: gardening. He planted, he nurtured, and he took care of and kept an eye on things; as the garden flourished, he kept it in bloom by careful but unobtrusive attention, preventive maintenance, and teaching and inspiring others to pitch in and help.

**Figure:**
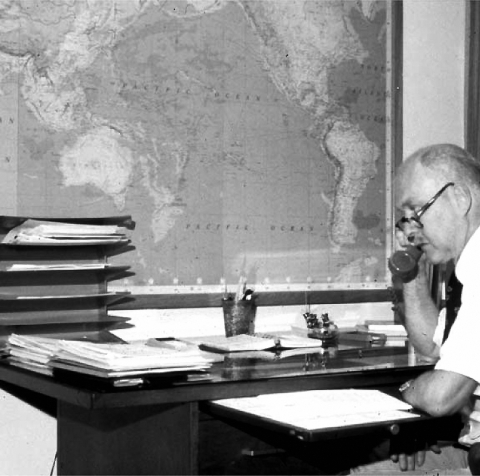
Mike Gregg at work on the Morbidity and Mortality Weekly Report. Source: Steve Thacker.

Mike’s textbook, Field Epidemiology (*2*), remains the authoritative work on outbreak investigations and the “shoe leather” approach to public health problem solving. It reflects not only his style but also his unparalleled experiences in national and international epidemiology. Between the mid-1960s and 1990, when he retired from CDC, Mike was often in the eye of the hurricane, presiding as deputy director over CDC’s Bureau of Epidemiology during many of the most memorable outbreak investigations of the past century. These included Pontiac fever/Legionnaires’ disease (1968/1976), swine flu (1975–1976), Guillain-Barré syndrome (1977), Ebola fever in Sudan and Zaire (1976), and AIDS (1981). He also helped put on the epidemiology map such new diseases as Reye syndrome (1973–1977), Kawasaki disease (1977), and toxic-shock syndrome (1980). And he helped steer us into an era of control (and future eradication) of such vaccine-preventable diseases as measles and poliomyelitis.

Less appreciated, his methods and perspectives led unself-consciously to the conceptualization and the development of approaches to understanding what would later be called emerging infectious diseases. He believed that such emergences were an inevitable consequence of microbial genetic dynamism let loose in ecosystems shared by humans and environmental determinants. If this were not enough, Mike described it all in the Morbidity and Mortality Weekly Report (MMWR), put out in the pithy, painfully unambiguous, “just-the-facts-ma’am” style he perfected. As an unintentional by-product of his editorial skills, he turned hundreds—perhaps thousands—of epidemiologists into decent medical writers and editors, instilling in them the idea that good writing is not only a result of, but also a path to, the kind of clear thinking needed in epidemiologic practice. Anne Mather, managing editor of the MMWR for 7 years under Mike’s leadership in the 1970s and 1980s, notes that his mantra for editing, “This is getting into the thick of thin things,” is advice she still follows, and quotes, today.

Even in full retirement, Mike was there for us to consult. When the outbreak of severe acute respiratory syndrome (SARS) erupted in 2003, Emerging Infectious Diseases called him into service to review a major report of a SARS investigation. Although he strongly recommended publication of the article, Mike’s 5-page review nonetheless respectfully took it apart line by line, then put it back together in the way it should have been done in the first place. The authors may have wished they had had this anonymous reviewer on their team during the actual investigation.

At CDC Mike avoided the limelight but devoted tremendous energy to supporting others, especially young epidemiology trainees (Epidemic Intelligence Service, or EIS, officers). When I first met him in 1975, he wore three tall hats: MMWR editor, Viral Diseases Division director, and deputy director of the Bureau of Epidemiology (this was 7 years before the “Center” became the “Centers,” a time when the “Bureau of Epi” was still CDC’s crown jewel). He was my first boss and already something of a legend. He taught us by making us apprentices in everything he did, including participation in countless “curbside” discussions of epidemics, methods, publications, theories, and epidemic history. The discussions were often conducted on the run, carried on from office to office like a movable feast, spilling into hallways, migrating to the cafeteria, or even the library. It always amazed me that such a quiet man was at the center of so much bustling activity.

Mike was also a historian of sorts, retaining much of the institutional memory of CDC. He had encyclopedic recall of past CDC outbreak investigations, perhaps because he had been involved in supervising many of them, reading and editing the “Epi-2” reports, and then publishing write-ups in the MMWR. The day we learned of an outbreak of respiratory disease among American Legionnaires in Philadelphia, Mike was having lunch in the CDC cafeteria with 3 young EIS officers (me among them), each of us less than a month on the job. Everyone was excited: obviously, something really big was happening. But, as is usually the case, information was dribbling in sporadically, incompletely, and with maddening imprecision. After listening to his young colleagues speculate a bit, Mike calmly reflected on whether this could be “the big one,” return of a 1918-like influenza pandemic presaged by the swine flu outbreak earlier that year. He ran through the old and the recent history of influenza and other epidemic respiratory diseases, calling up all sorts of experiences completely new to us, commenting on what we (Pennsylvania health officials and CDC) needed to look for, what facts would be for and against influenza, what we needed to do next, and so on. I was thrilled. On ward rounds only a few weeks beforehand, I was now suddenly in the middle of a national epidemic, having lunch with one of the nation’s top epidemiologists, learning about fascinating things I had not imagined last month. “Yes!” I thought, “This epidemiology thing is what I want to do from now on.” Many others had similar epiphanies.

Within a day or two of that lunch discussion, accumulating information made it clear that the Philadelphia outbreak could not be influenza. Remembering a mysterious unsolved epidemic 8 years beforehand, and having himself caught that mystery disease in the line of duty, Mike was then perhaps the first to say “This is beginning to look like Pontiac fever.” And so it turned out to be.

Each July, Mike addressed the incoming class of EIS officers, typically fresh from the wards of Ivy League medical schools, in the only epidemiology course most would ever attend. His first subject would be “how to investigate an epidemic,” one of the few medical subjects most of his audience knew absolutely nothing about. Peering up shyly over his glasses, held midway down the bridge of his nose, he would look down at the floor for a minute and then begin so softly that those in the back would have to strain to hear. “First,” he would say, “you need to find a good map...” Then, in a slow build-up of pragmatic and mundane steps, he would walk us through an outbreak, taking care to omit none of those boringly essential details that seemed too obvious to mention. His unstated message was clear and powerful. This isn’t medical school. Memorizing the Krebs cycle, the cranial nerves, and the entire Washington Manual won’t get you through an outbreak investigation. You are now a detective, and you have to go about investigating the scene of the “accident” in “real time,” with little opportunity to research or look things up, keeping an open and inquisitive mind, remaining flexible and creative, always rethinking things, backtracking and verifying when necessary, being thorough but also moving quickly to assemble all of the puzzle pieces that seek to become a coherent picture.

“Quick and dirty,” one of his favorite terms, was how he described much of what we epidemiologists did, and not in a pejorative sense of the word but with genuine affection for the game of hide-and-seek he never tired of playing. In epidemiologic investigations, there was no time for elegance and perfection. Epidemiology helped real people with real problems. As Michael O’Leary, another of Mike’s former epidemiology students, paraphrased him: “It’s better to be approximately right today than exactly right tomorrow.” Were Mike Gregg standing in Golden Square in 1854, he would have been impressed less by John Snow’s brilliant reasoning than by Snow’s ability to get the Broad Street pump handle removed quickly, thereby saving more lives.

Avuncular, almost fatherly at times, Mike clearly enjoyed mentoring young colleagues, and in doing so he revealed to us a broader view of a bigger, more complicated world into which we might one day be admitted. His door was always open, no matter how low on the totem pole you might be; but if he needed to see you, he popped into your office rather than asking you to come to his. In conversation he valued and respected the ideas of even the newest of neophytes. He listened more than he spoke; and when he did speak, it was softly and courteously, tentatively offering up his own thoughts on the subject, as if this were a democratic effort, rather than the boss telling you how things really were. He embraced teamwork as the professional norm and ignored, without discounting or disparaging it, individual accomplishment. His modesty was genuine; his gentleness, memorable. He walked the halls with his head aimed shyly down, moving so close to the cinderblock wall that you expected him to bump into it at any moment, occasionally lifting his shoulder to avoid just such an accident, dressed casually and in a manner that never telegraphed his stature, his slightly battered Hush Puppies pressing silently on linoleum floors. Had you passed Mike in the cafeteria, or even sat down next to him at a meeting, you might have mistaken him for a visitor who had accidentally wandered into Building 1.

Mike’s conversation was sometimes reflective and philosophical but at other times spare, to the point, and without adornment. Frequently, before speaking he softly cleared his throat, as if to test that he had enough voice to be heard, or perhaps as a preventive warning against interrupting someone else. If he became irritated or impatient, which happened only rarely, there might appear a quick gleam in his eyes, but any anger he felt dissipated quickly. In more than 30 years, I never heard him raise his voice above a moderate level. When others drowned him out, he let them do so, dropping his own words to listen with interest and respect to theirs.

His sense of humor was expressed not in guffaws but in short chuckles, sometimes accompanied by the split-second flash of an almost-mischievousness grin. Like everything else about him, his humor was understated. One example comes to mind. Gracious hosts, he and his wife, Lila, sometimes opened their home to the annual picnics for incoming EIS officers and their families. On the first of these I attended, in 1976, I remember him relating, with a seriousness that may well have been intentionally hilarious (I could never be sure), the story of a scandalous, and possibly apocryphal, EIS-picnic-from-hell of some years past. In Mike’s telling, someone on the CDC staff had gotten a not-so-bright idea for a training exercise: put methylene blue dye in one of the foods at the EIS picnic and, when the results were discovered, send the EIS officers off on an outbreak investigation to identify the food source. Apparently, however, when spouses and children began urinating blue liquid into their home toilets, a few among them were more alarmed than amused, and the exercise had to be abandoned. Mike told this story dryly but with a twinkle in his eye, as if to say “What a dumb thing to do (but wasn’t it a great idea for an epidemic—more than ‘eleven blue men’ [*3*] but women and children too?”).

One of my most vivid memories of Mike concerns a brief discussion we had in about 1978. I was sitting outside the office of Phil Brachman, then the director of the Bureau of Epidemiology, when Mike walked up. Unexpectedly, he greeted me in French, “Ça va”?. Remembering his Parisian birth, I replied in kind, then asked how he had come to be born in France. His father had worked there at the Rockefeller’s European office, he said, continuing on with a few remembrances about the man. I had vaguely remembered hearing about his famous father, but Mike’s recollections were about a beloved “Dad,” not a famous man. It was some years later that I learned who Alan Gregg was—a legendary Rockefeller Foundation official who made a tremendous and lasting impact on biomedical research—but I never forgot the touching sense of fondness and reverence with which Mike spoke about his dad that day.

At age 78, Mike Gregg died too young and too quickly for many to grasp the meaning and consequences of his passing. His touch was so light that it is difficult to comprehend the breadth and depth of his legacy or to measure his influence on epidemiology and American public health. He would surely take quiet pride in being warmly remembered as the quintessential epidemiologist by friends and colleagues around the world. But I imagine he would be prouder still to know that his gardening was a success, that the garden still flourishes, and that it is now tended by those who learned from him and will one day share his secrets with others.
